# Poorer Quality of Life and Treatment Satisfaction is Associated with Diabetic Retinopathy in Patients with Type 1 Diabetes without Other Advanced Late Complications

**DOI:** 10.3390/jcm8030377

**Published:** 2019-03-18

**Authors:** Minerva Granado-Casas, Esmeralda Castelblanco, Anna Ramírez-Morros, Mariona Martín, Nuria Alcubierre, Montserrat Martínez-Alonso, Xavier Valldeperas, Alicia Traveset, Esther Rubinat, Ana Lucas-Martin, Marta Hernández, Núria Alonso, Didac Mauricio

**Affiliations:** 1 Department of Endocrinology and Nutrition, Health Sciences Research Institute & University Hospital Germans Trias i Pujol, 08916 Badalona, Spain; mgranado@igtp.cat (M.G.-C.); esmeraldacas@gmail.com (E.C.); aramirez@igtp.cat (A.R.-M.); nalonso32416@yahoo.es (N.A.); 2 Lleida Institute for Biomedical Research Dr. Pifarré Foundation, IRBLleida, University of Lleida, 25198 Lleida, Spain; cubito@lleida.org (N.A.); martahernandezg@gmail.com (M.H.); 3 Centre for Biomedical Research on Diabetes and Associated Metabolic Diseases (CIBERDEM), Instituto de Salud Carlos III, 08907 Barcelona, Spain; 4 Department of Endocrinology & Nutrition, University Hospital Germans Trias i Pujol, 08916 Badalona, Spain; marionamarting@gmail.com (M.M.); alucas.germanstrias@gencat.cat (A.L.-M.); 5 Systems Biology and Statistical Methods for Biomedical Research, IRBLleida, University of Lleida, 25198 Lleida, Spain; mmartinez@irblleida.cat; 6 Department of Ophthalmology, University Hospital Germans Trias i Pujol, 08916 Badalona, Spain; xvalldeperas@gmail.com; 7 Department of Ophthalmology, University Hospital Arnau de Vilanova, 25198 Lleida, Spain; atravesetm@gmail.com; 8 Department of Nursing and Physiotherapy, University of Lleida, 25198 Lleida, Spain; rubinatesther@gmail.com; 9 Department of Endocrinology & Nutrition, University Hospital Arnau de Vilanova, 25198 Lleida, Spain; 10 Department of Endocrinology & Nutrition, Hospital de la Santa Creu i Sant Pau, 08041 Barcelona, Spain

**Keywords:** diabetic retinopathy, type 1 diabetes, quality of life, treatment satisfaction, patient-reported outcomes

## Abstract

Diabetic retinopathy (DR) may potentially cause vision loss and affect the patient’s quality of life (QoL) and treatment satisfaction (TS). Using specific tools, we aimed to assess the impact of DR and clinical factors on the QoL and TS in patients with type 1 diabetes. This was a cross-sectional, two-centre study. A sample of 102 patients with DR and 140 non-DR patients were compared. The Audit of Diabetes-Dependent Quality of Life (ADDQoL-19) and Diabetes Treatment Satisfaction Questionnaire (DTSQ-s) were administered. Data analysis included bivariate and multivariable analysis. Patients with DR showed a poorer perception of present QoL (*p* = 0.039), work life (*p* = 0.037), dependence (*p* = 0.010), and had a lower average weighted impact (AWI) score (*p* = 0.045). The multivariable analysis showed that DR was associated with a lower present QoL (*p* = 0.040), work life (*p* = 0.036) and dependence (*p* = 0.016). With regards to TS, DR was associated with a higher perceived frequency of hypoglycaemia (*p* = 0.019). In patients with type 1 diabetes, the presence of DR is associated with a poorer perception of their QoL. With regard to TS, these subjects also show a higher perceived frequency of hypoglycaemia.

## 1. Introduction

Diabetic retinopathy (DR) is a significant diabetic complication in patients with type 1 diabetes [1]. This complication is a potential cause of vision loss in the diabetic population that can negatively affect their quality of life (QoL) [2]. Moreover, DR is also associated with an increased risk for all-cause mortality and cardiovascular disease in patients with type 1 diabetes [3].

QoL is a multidimensional, subjective and dynamic construct comprising the individual’s subjective perception of the physical, psychological and social well-being aspects of his or her life [4]. Treatment satisfaction (TS) is an individual’s subjective appraisal of his or her experience of the treatment, including both the process and results [4]. From the patient’s point of view, QoL and TS as patient-reported outcomes (PROs) are two important measures because these are often stronger determinants of medical outcomes such as hospitalization, mortality and the presence of complications [5,6]. Furthermore, an assessment of PROs has been accepted to complement visual acuity information in clinical trials, interventions or research [7]. Regarding QoL and TS, recent studies have found that having DR negatively impacts the QoL and TS in patients with type 2 diabetes [8,9,10,11,12,13,14].

To our knowledge, only one study with a cross-sectional design has assessed the relationship between QoL and the presence of DR in patients with type 1 diabetes using a diabetes-specific QoL questionnaire [15]. So far, all of the published scientific evidence regarding this issue used generic instruments or visual function scales to appraise the impact of DR in the QoL of patients with type 1 diabetes [16,17,18,19,20,21,22,23,24,25,26,27,28,29,30]. The FinnDiane study, which included a large sample of patients with type 1 diabetes, did not find any association between the presence of DR and health-related quality of life (HRQoL) using a generic instrument [16]; it should be pointed out that, in this study, DR was determined to be present if the subject had ever received treatment for DR and that 53% of the patients had also diabetic nephropathy, which could influence the results. Other cross-sectional studies with large samples of patients with type 1 diabetes did not report any association between DR and HRQoL [17,23,24]. However, other prospective cohort and cross-sectional studies observed a negative impact of DR on HRQoL in patients with type 1 diabetes using generic instruments [25,28]. Two recently published studies found a lower HRQoL in patients with type 1 diabetes and more severe DR in the presence of other diabetic complications such as neuropathy, nephropathy and cardiovascular disease [29,30]. The Wisconsin Epidemiologic Study of Diabetic Retinopathy did not find changes in the QoL with the presence of DR over time using a visual function scale [18,19]; nevertheless, researchers included a study sample of patients with other important diabetic complications that could influence the results. Furthermore, the Diabetes Control and Complications Trial/Epidemiology of Diabetes Interventions and Complications (DCCT/EDIC) Study designed to assess the effects of intensive insulin treatment and risk factors on the patient-reported visual function in patients with type 1 diabetes did not find changes with DR [20]. Although researchers used a diabetes-specific QoL questionnaire and a visual function scale, these patients also showed other diabetic complications. Other studies performed in patients with type 1 and type 2 diabetes using a visual function scale related lower scores with DR [21,22]; however, the study subjects had also other diabetic complications and comorbidities such as cardiovascular diseases, cancer, arthritis or rheumatism and hearing problems. Regarding DR and PROs, it has been pointed out that generic instruments have a lack of sensitivity to assess specific domains of QoL that are related to diabetic complications [7]. This is an important point because the scientific evidence has shown that patients can report a good patient-reported health status, often measured with generic questionnaires, even though their diabetes-specific QoL is negative [8,9,31]. Besides, visual function scales cannot be regarded as sufficient to assess the diabetes-related QoL due to the lack of specific quality of life domains related to diabetes. For this reason, the disease-specific questionnaires, but not the generic tools, are the more adequate instruments to measure specific disease-related QoL [31].

To the best of our knowledge, there have not been any studies primarily designed to assess the impact of DR on QoL and TS in patients with type 1 diabetes. In addition, there are not any studies that have assessed the impact of DR on QoL and TS that included a well-defined sample of patients with type 1 diabetes and that used diabetes-specific questionnaires. Therefore, we hypothesized that patients with type 1 diabetes who have been diagnosed with DR have a lower perception of their QoL and TS in comparison to their non-DR counterparts. The aim of the study was to assess the association of DR with QoL and TS in adult type 1 diabetic patients with DR as compared to a group of patients without this condition. Additionally, we also determined which clinical factors that could be related to QoL and TS.

## 2. Materials and Methods

This was a cross-sectional, two-centre study. Participants were patients with type 1 diabetes regularly cared for at their reference hospital from two health care regions (Lleida and Badalona). The recruitment took place between January 2013 and May 2015. From a total number of 330 patients with type 1 diabetes that were invited to participate, 276 accepted to be included in the study, while 17 could not be included because of exclusion criteria. Finally, a total sample of 259 patients with type 1 diabetes was included in the previously published study [32]. From this sample, 16 patients were excluded because they had not had their eyes assessed; furthermore, one patient was excluded because he did not answer the questionnaires. A final sample of 102 patients with DR and 140 without DR were included. A detailed description of the characteristics of the study are provided elsewhere [32]. The inclusion criteria were: having the diagnosis of type 1 diabetes, a current age greater than 18 years, and a disease duration of more than 1 year. We intended to avoid the inclusion of other advanced late diabetic complications to exclude their negative impact on top of DR. Thus, the exclusion criteria were as follows: psychological or cognitive deterioration (e.g., mental diseases or dementia); being a healthcare professional; the presence of previous clinical cardiovascular diseases (ischemic heart disease, cerebrovascular disease, peripheral arterial disease and heart failure) or diabetic foot disease; the presence of any eye disease that could influence the results (macular oedema, media opacity that hindered assessment of the retina, other concomitant retinal pathology, ophthalmological surgery in the previous year and laser treatments during the six months before the study visit); pregnancy; and the presence of other advanced diabetic late complications such as macroalbuminuria (urine albumin/creatinine ratio > 299 mg/g) and renal insufficiency (estimated glomerular filtration rate < 60 mL/min). In the DR group, three patients had glaucoma and one patient had myopia superior to 5 dioptres. In the non-DR group, three patients had myopia superior to 5 dioptres. The assessment of DR was performed and classified by an expert ophthalmologist according to the international clinical classification system [33]. Optic coherence tomography measurements were performed using spectral-domain OCT (SD-OCT) (Cirrus HD-OCT, model 4000, Carl Zeiss Meditec, Dublin, CA, USA) and deep Enhanced Imaging (EDI-OCT), with HD 5 Line Raster scan pattern. An ophthalmologist assessed and classified DR as follows: no apparent retinopathy, mild non-proliferative retinopathy, moderate non-proliferative retinopathy, severe non-proliferative retinopathy and proliferative retinopathy. The ethics committee of each of the two participating centres (Ethics Committee of the University Hospital Arnau de Vilanova, and Ethics Committee of the University Hospital Germans Trias i Pujol) approved the study. Written informed consent form was obtained from all participants.

### 2.1. Clinical Variables

Anthropometric measures, including waist circumference, weight, height and body mass index (BMI), blood pressure and laboratory variables were determined according to standard procedures. Cardiovascular disease was excluded based on detailed anamnesis and careful review of all clinical records. Besides, predefined questionnaires specially designed for the study were used for conduction of personal interviews and data extraction for collection of all other variables: medication use, physical activity, educational level, ethanol consumption and smoking habit. Hypertension and dyslipidaemia were defined by the use of specific medication for treatment of these two conditions. Microalbuminuria was defined as an albumin creatinine ratio above 30 mg/g. Physical activity was assessed according to two previously validated methods [34,35]. This was classified as regular physical activity (if the patients spent more than 25 min/day in any activity that requires 4 METS (the metabolic equivalent), or as sedentary if the participants spent up to 25 min/day. Ethanol consumption (g per day) was estimated from frequency, beverage type, and average amount according to previously validated methods [32].

### 2.2. Quality of Life

The Audit of Diabetes-Dependent Quality of Life (ADDQoL-19) questionnaire in Spanish was used to assess the QoL of the patients. This is an instrument specifically designed for its use in subjects with diabetes, and validated in the diabetic Spanish population [36,37,38]. The questionnaire consists of 21 items, 19 of which are related to specific life domains and are scored multiplying the impact rating (from −3 to +1) by the importance rating (from +3 to 0); these produce scores ranging from +3 to −9 points. The overall score is the mean of 19 specific domains and is assigned as the average weighted impact score (AWI); this ranges from +3 (highest QoL) to −9 (poorest QoL). Additionally, there are two overview items that are scored separately; they measure present QoL ranging from +3 (excellent) to −3 (very bad) and diabetes-specific QoL, which is scored from −3 (maximum negative impact) to +1 (maximum positive impact). Two trained researchers (MG-C and MM) conducted individual interviews with all of the patients. All questionnaires have been recommended by the World Health Organization and the International Diabetes Federation to assess the PROs [6].

### 2.3. Treatment Satisfaction

The Diabetes Treatment Satisfaction Questionnaire-status version (DTSQ-s) in Spanish, a diabetes-specific questionnaire validated for Spanish diabetic subjects was administered [39,40]. This questionnaire consists of 8 items scored on a 6-point scale from 0 (very unsatisfied) to 6 (very satisfied). The final score is calculated by summing up the individual scores from six items (current treatment, convenience, flexibility, understanding, recommend to others and continue with); this final score ranges from 36 (very satisfied) to 0 (very unsatisfied). Additionally, two items are calculated separately and measure the perceived frequency of hyperglycaemia and hypoglycaemia, respectively; they are scored from 0 (never) to 6 (always).

### 2.4. Sample Size

The sample size was determined by using the standard deviation for the ADDQoL items from a previously published study on DR and QoL that was performed in patients with type 2 diabetes by our group in the same region [8]. Then, we assumed that the differences would be similar in patients with type 1 diabetes for present QoL, diabetes-specific QoL and AWI items. A sample of 54 patients with DR and 54 without DR was deemed necessary to detect significant differences between both groups. We used the Mann-Whitney test and a statistical power of 80% with a significance level of 5%. From our previous study, a sufficiently large number of patients with type 1 diabetes was available to fulfil the sample size [32].

### 2.5. Statistical Methods

The descriptive analysis of quantitative variables included mean and standard deviation for the normally distributed variables, as well as median and interquartile intervals. Qualitative variables were summarized by absolute and relative frequencies. Bivariate analysis comparing the groups of patients with and without diabetic retinopathy included the *t*-test (or the Mann–Whitney test for non-normally distributed variables) and the chi-squared test for quantitative and qualitative patient characteristics as well as QoL and TS outcomes (including overall and subscale scores), respectively. Simple linear regression analysis was performed to assess the univariate association of patients’ characteristics with overall QoL and TS, as well as each of their domains. Those domains showing a significant univariate association with DR were evaluated in a linear multivariable regression analysis to assess the statistical significance of their association after having adjusted by other patients’ characteristics. For this purpose, in a first step and separately for each domain associated with DR, we estimated a multivariable linear (or ordinary least squares) regression model including all variables with a *p*-value <0.25 according to the likelihood ratio test (LRT). In the next step, we used the same method (LRT) to simplify the model by dropping non-significant variables, starting with the one with the highest and non-significant *p*-value, until obtaining a model with variables showing a significant contribution according to the LRT. Afterwards, we checked the possible additional significant contribution of any other patient characteristics. The final proposed regression models included any possible interactions involving the patient characteristics that were included in the corresponding regression model. We used a significance level of 0.05 and the open-source program R for all statistical analysis [41].

## 3. Results

The clinical and demographic characteristics of the participants are shown in Table 1. Patients with DR were older (*p* = 0.004), had higher systolic blood pressures (*p* = 0.003), higher glycated haemoglobin (HbA1c) (*p* < 0.001), longer diabetes duration (*p* < 0.001) and lower high density lipoprotein (HDL) concentrations (*p* = 0.015) in comparison with non-DR patients. Furthermore, the former had a higher frequency of hypertension (*p* < 0.001) and microalbuminuria (*p* = 0.014).

### 3.1. Diabetes-Related Quality of Life

Patients with DR showed lower present QoL (*p* = 0.039) in comparison with their counterparts without DR (Table 2). Work life and dependence scores were also lower in patients with DR (*p* = 0.037 and *p* = 0.010, respectively). Although no differences between the groups in the diabetes-specific QoL item (*p* = 0.069) were observed, the AWI score showed a poorer QoL in patients with DR (*p* = 0.045). In the multivariable analysis, DR was associated with a low score in present QoL (β = −0.25; *p* = 0.040) and with a poorer perception of QoL items such as work life (β = −0.78; *p* = 0.036) and dependence (β = −0.83; *p* = 0.016) (Table 3). Together with smoking habit, these variables only explained a 2.0%, 2.3% and 3.8% of the variability of those scores, respectively. On the other hand, no association was found between DR and the AWI score (*p* = 0.111) after adjusting for smoking status, age, insulin dose and physical activity (Supplemental Table S1). However, there was a negative correlation between older age and the AWI score (*p* = 0.015). Conversely, physical activity and the daily insulin dose showed a positive association with a higher AWI score (*p* = 0.005 and *p* = 0.028, respectively).

To further explore the effect of advanced DR stages on QoL and TS, we analyzed differences between study groups: without DR, with mild DR (grade 1), and more than mild DR (grades 2–4) (Supplemental Table S2). The comparison among the 3 groups revealed no statistical differences. However, head to head comparison between groups yielded some differences. For instance, patients with mild DR showed a poorer perception of the present QoL (*p* = 0.040), dependence (*p* = 0.005), and an AWI score (*p* = 0.038) in comparison with non-DR patients (Supplemental Table S2). Furthermore, patients with advanced (more than mild) DR had a lower AWI score (*p* = 0.032) in comparison with the non-DR group. No statistical differences were observed between groups with the other items (Supplemental Table S2).

### 3.2. Treatment Satisfaction

The perception of the frequency of hypoglycaemia was higher in the DR group (*p* = 0.022) (Table 2). However, no significant differences were observed between the groups in terms of hyperglycaemia frequency perception and the DTSQ-s final score. We did not find any difference among groups defined by the status of DR in any of the DTSQ-s items (Supplemental Table S2). In the multivariable analysis for the hypoglycaemia frequency perception item of the DTSQ-s, we observed a higher hypoglycaemia frequency perception in subjects with DR (β = 0.019; *p* = 0.019) (Table 4). In addition, male sex was associated with a lower hypoglycaemia frequency perception (β = −0.60; *p* = 0.001). DR together with male sex only explained 6.3% of the variability. In Supplemental Table S3, the multivariable analysis of the DTSQ-s final score did not show any relationship with DR (*p* = 0.244). Only sex and diabetes duration were associated with the DTSQ-s final score.

## 4. Discussion

Our results indicate that patients with type 1 diabetes and DR had a poorer QoL than those without DR. In addition, DR is associated with poorer individual items related to QoL, i.e., present QoL, work life and dependence. Additionally, older age was also associated with a poorer QoL; however, physical activity and insulin dose were associated with a higher QoL. In terms of overall TS, although no difference between the two groups was observed, DR was associated with a higher hypoglycaemia frequency perception. In addition, a longer diabetes duration in men was associated with higher TS.

The current study showed a poorer perception of the QoL in the presence of DR; this could be due to the fact that DR is a condition that more frequently develops in those patients with poorer management of the disease and less healthy lifestyle behaviour. This could explain, at least in part, the poorer perceived QoL of patients with DR in comparison with those without DR. However, there is only one previous cross-sectional study that has assessed the QoL in patients with type 1 diabetes using a diabetes-specific QoL instrument [15]. That study did not find any association between DR and QoL, which is in contrast with our results. Nevertheless, this could be because patients in that study had a high frequency of advanced late complications, such as diabetic foot disease, which could be potential confounders. In addition, the previous study was not specifically designed to assess the relationship between DR and QoL; the authors studied a sample of patients with type 1 and type 2 diabetes together, with a small sample of patients with DR (*n* = 74) [15]. Other cross-sectional studies that focused on patients with type 1 diabetes and used generic instruments to measure QoL also did not find an association between DR and QoL [16,17,24]. Fenwick et al. [23] performed a cross-sectional study to assess the impact of DR on the QoL with a sample of patients that included both type 1 and type 2 diabetes. They did not find any association between DR and QoL because they used a generic instrument; besides, these cross-sectional studies were not specifically designed to assess the relationship between the QoL and DR in this population. Nevertheless, a prospective study performed on patients with type 1 diabetes using generic instruments found a negative relationship between the presence of late diabetic complications and HRQoL [25]. However, these patients had a higher frequency of comorbidities and other complications apart from DR. Peasgood et al. [28] conducted a post hoc analysis from an interventional study and observed a decreased HRQoL in patients with type 1 diabetes who also had DR. These results are consistent with the results of the current study. Although they also used generic instruments to measure a specific condition associated with diabetes, the sample showed other diabetic complications, which were self-reported; all of these characteristics could influence the results. Furthermore, another cross-sectional study involving patients with type 1 diabetes observed a lower HRQoL in patients with severe DR; this is similar to our findings, although their results were not adjusted for other complications [29]. There are two cross-sectional studies performed with a sample of patients with type 1 and type 2 diabetes that found a lower HRQoL in the presence of DR [26,27]. These are similar findings to the current study, even though the authors used generic instruments and data from both types of diabetes were pooled. A previous study performed on patients with type 2 diabetes by our group showed the negative impact of the presence and severity of DR on the QoL [8]. Additionally, the PANORAMA study, which was conducted with a large sample of patients with type 2 diabetes, also showed that a negative perception of QoL was associated with the presence of DR [9]; this is similar to the current results in patients with type 1 diabetes, even though the two types of diabetes have remarkably different characteristics. There are other studies that yielded results that are discordant with our findings. First, the Wisconsin Epidemiologic Study for Diabetic Retinopathy did not find changes in the QoL with the presence and severity of DR [18,19]. We should point out that in that study the authors used a visual function scale to assess the diabetes-specific QoL. Furthermore, a relevant proportion of the study subjects had other diabetic complications that could significantly impact on QoL, such as nephropathy, neuropathy, cardiovascular diseases and limb amputations. Finally, the DCCT/EDIC study did not observe a relationship of QoL with DR [20]; although they used a diabetes-specific instrument and a visual function scale, again the study subjects showed other complications that could influence QoL.

The factor associated with a poorer QoL was advanced age; this is in line with our previous results in subjects with type 2 diabetes [42]. Nyanzi et al. [15] found a negative relationship between age and some aspects of QoL, which is similar to our results; moreover, in line with our study, they did not find any association between smoking status and perceived QoL. Other studies performed in patients with type 1 and type 2 diabetes also found that a reduced QoL was associated with older age [9,24,43,44]. However, a cross-sectional study of patients with type 2 diabetes did not find any association between QoL and these factors, despite using a diabetes-specific QoL instrument [14]. On the other hand, physical activity was associated with a higher QoL in patients with type 1 diabetes, which is in line with our previously published results about patients with type 2 diabetes [8,42]. However, we could not identify any study that has specifically assessed this issue in the diabetic population. The association of the daily insulin dose with a higher QoL has not been described in type 1 diabetes. We have no clear explanation for this finding. Lastly, emotional factors, such as the lack of optimism and a depressive style, contribute to impairing the QoL in women with type 1 diabetes [44]. However, in the current study we did not find any statistical difference between men and women in the emotional items of the QoL questionnaire.

In the current study, the DTSQ-s final score was neither different between the two study groups nor correlated with the presence of DR; this is similar to the results of the recent PANORAMA study and other studies performed on patients with type 2 diabetes [8,9,45]. However, no studies have assessed the relationship between DR and TS in patients with type 1 diabetes specifically. Furthermore, we found that DR was associated with a higher hypoglycaemia frequency perception. This increased perception of hypoglycaemia is probably associated with poorer glycaemic control, which is associated with a high risk for the development of diabetic complications such as DR [3]. Another factor that may influence this perception in patients with type 1 diabetes is ethanol consumption. However, there were no differences between both study groups in ethanol intake. A longer diabetes duration in men was associated with higher TS and a lower hypoglycaemia frequency perception; this is also in line with our previous results [8,42] and with other cross-sectional studies performed in patients with type 2 diabetes [9,45].

This study has several limitations. A causality association between QoL, TS and DR in patients with type 1 diabetes cannot be established due to the cross-sectional study design; additionally, changes over time in terms of QoL and TS cannot be determined by this study. In addition, hypoglycaemia unawareness and the frequency of self-blood glucose monitoring are factors that may influence the hypoglycaemia frequency perception in patients with type 1 diabetes. The absence of data on these aspects in our study is another limitation. Furthermore, it is well-known that insulin pump therapy is a treatment option for those patients with recurrent episodes of severe hypoglycaemia. Therefore, this treatment option may also have an impact on the perception of hypoglycaemia frequency; unfortunately, although the proportion of patients treated with pump therapy is low in our region, we did not collect data on this mode of therapy. Unfortunately, in the current study we did not assess the visual function of the study participants. In this regard, we should point out that the impact of DR on QoL is mainly produced by impairment of the visual function. Furthermore, although we could analyse the effect of advanced DR stages, the number of patients with advanced DR (moderate, severe and proliferative DR) was very low; for this reason, we cannot draw any conclusion as to whether patients with more severe DR are those than have poorer QoL and treatment satisfaction outcomes. This also applies to conditions known to produce visual impairment (e.g., myopia) that were present in a low number of participants. Although DR was significantly associated with specific QoL domains and overall diabetic treatment satisfaction, according to our results, there is a high percentage of variability in these patient-reported outcomes that remains unexplained. Actually, some other factors or comorbidities associated with DR, that were not assessed in the current study, could have a relevant contribution to explain our findings on QoL and TS. For instance, although we used diabetic foot disease as an exclusion criterion, we did not assess diabetic neuropathy in this sample of patients, a complication that could affect the QoL and TS of the patients. All the limitations mentioned above call for caution when interpreting the current results. Further studies are clearly needed to address all the aforementioned issues, especially those in relation to eye-related burden, i.e., the contribution of the impairment of visual function and the impact of advanced stages of DR.

On the other hand, this study has several strengths. This is the first study that has assessed the potential relationship between QoL and TS in adult patients with type 1 diabetes and DR by using diabetes-specific QoL and TS questionnaires. Many studies have used generic instruments to assess QoL. In point of fact, generic instruments cannot assess the impact of a specific condition on the aspects of patient’s life due to a demonstrated lack of sensitivity of these instruments; this has been described in scientific literature [5,6,7]. Additionally, we recruited a relevant number of patients with type 1 diabetes without other diabetic complications and comorbid conditions in this two-centre study and included a representative sample of subjects.

## 5. Conclusions

In conclusion, DR was associated with poorer QoL and TS in patients with type 1 diabetes. Moreover, DR was associated with a higher hypoglycaemia frequency perception. Finally, the current results have implications for potential future research because there has been no other study that specifically assesses the impact of DR on the QoL and TS of adult patients with type 1 diabetes. Future research should lead to the identification of the individual contribution of each of the relevant factors on these patient-reported outcomes.

## Figures and Tables

**Table 1:** Clinical and demographic characteristics of the study groups.

Characteristics	No DR (*n* = 140)	DR (*n* = 102)	*p*
Age (years)	42.1 ± 10.3	46.2 ± 10.8	0.004
Sex (men)	62 (44.3)	47 (46.1)	0.884
Race (Caucasian)	137 (97.9)	101 (99.0)	0.640
Study site			0.168
Badalona	85 (60.7)	52 (51.0)	
Lleida	55 (39.3)	50 (49.0)	
Educational level			0.069
Less than primary school	8 (5.9)	4 (4.1)	
Completed primary school	34 (25.2)	35 (36.5)	
Secondary/high school	49 (36.3)	39 (40.6)	
Graduate school or higher	44 (32.6)	18 (18.8)	
Smoking			0.282
Non or former smoker	106 (75.7)	70 (68.6)	
Yes	34 (24.3)	32 (31.4)	
Regular physical activity	98 (70.0)	77 (75.5)	0.425
Insulin dose (UI/kg/day)	0.57 [0.41; 0.75]	0.65 [0.45; 0.86]	0.027
Waist circumference (cm)	86.0 [79.0; 95.0]	90.0 [81.0; 100.0]	0.050
Systolic blood pressure (mmHg)	120.0 [110.0; 134.0]	130.0 [118.0; 140.0]	0.003
Diastolic blood pressure (mmHg)	74.2 ± 9.6	73.6 ± 9.3	0.666
Body mass index (kg/m^2^)	24.7 [22.4; 27.3]	25.9 [23.1; 28.3]	0.091
Hypertension	20 (14.3)	40 (39.2)	<0.001
Dyslipidaemia	49 (35.0)	48 (48.0)	0.056
Microalbuminuria	5 (3.6)	13 (12.9)	0.014
Diabetes duration (years)	17.0 [11.0; 22.0]	26.5 [19.2; 33.0]	<0.001
Glaucoma	–	3 (2.9)	–
Myopia over 5 dioptres	3 (2.1)	1 (0.9)	–
HbA1c (%)	7.3 [6.8; 7.8]	7.7 [7.2; 8.5]	<0.001
HbA1c (mmol/mol)	56.3 [50.8; 61.7]	60.7 [55.2; 69.4]	<0.001
Total cholesterol (mg/dL)	177.0 [164.0; 201.0]	176 [158.0; 200.0]	0.560
HDL cholesterol (mg/dL)	65.5 [55.0; 75.0]	59.5 [51.0; 71.0]	0.015
LDL cholesterol (mg/dL)	102.0 [87.0; 115.0]	100.0 [83.2; 119.0]	0.777
Triglycerides (mg/dL)	64.0 [49.8; 80.0]	68.0 [53.0; 89.8]	0.084
Ethanol consumption (g/day)	5.6 ± 9.2	6.5 ± 11.6	0.686

Data are shown as median [interquartile], means ± SD or *n* (%). DR, diabetic retinopathy; HbA1c, glycated haemoglobin; HDL, high density lipoprotein; LDL, low density lipoprotein.

**Table 2:** Bivariate analysis for the Audit of Diabetes Dependent Quality of Life (ADDQoL-19) and the Diabetes Treatment Satisfaction Questionnaire-status (DTSQ-s) by diabetic retinopathy status.

Items	No DR (*n* = 140)	DR (*n* = 102)	*p*
ADDQoL-19			
Present QoL	1.00 [1.00; 2.00]	1.00 [0.00; 1.00]	0.039
Diabetes-specific QoL	−1.00 [−2.00; −1.00]	−2.00 [−2.00; −1.00]	0.069
Leisure	−1.00 [−3.00; 0.00]	−2.00 [−3.75; 0.00]	0.198
Work life	0.00 [−3.00; 0.00]	−2.00 [−4.00; 0.00]	0.037
Travel	−2.00 [−4.00; 0.00]	−1.00 [−3.75; 0.00]	0.242
Holidays	−2.00 [−3.00; 0.00]	−2.00 [−3.00; 0.00]	0.987
Physical ability	−2.00 [−4.00; 0.00]	−2.00 [−4.00; 0.00]	0.306
Family life	0.00 [−3.00; 0.00]	0.00 [−3.00; 0.00]	0.333
Friends/social life	0.00 [0.00; 0.00]	0.00 [0.00; 0.00]	0.556
Personal relationships	0.00 [−2.00; 0.00]	0.00 [−2.00; 0.00]	0.122
Sex life	0.00 [−2.00; 0.00]	0.00 [−3.00; 0.00]	0.054
Physical appearance	0.00 [−1.00; 0.00]	0.00 [−2.00; 0.00]	0.144
Self-confidence	0.00 [−2.25; 0.00]	0.00 [−3.75; 0.00]	0.315
Motivation	0.00 [−2.00; 0.00]	0.00 [−2.00; 0.00]	0.950
Society/people’s reactions	0.00 [0.00; 0.00]	0.00 [0.00; 0.00]	0.301
Future	−2.00 [−4.00; 0.00]	−2.00 [−6.00; 0.00]	0.144
Finances	0.00 [0.00; 0.00]	0.00 [0.00; 0.00]	0.070
Living conditions	0.00 [0.00; 0.00]	0.00 [0.00; 0.00]	0.853
Dependence	0.00 [−3.00; 0.00]	−2.00 [−4.00; 0.00]	0.010
Freedom to eat	−4.00 [−6.00; −2.00]	−4.00 [−9.00; −2.00]	0.447
Freedom to drink	−2.00 [−4.50; 0.00]	−2.50 [−6.00; 0.00]	0.270
AWI	−1.32 [−2.05; −0.68]	−1.61 [−2.66; −0.94]	0.045
DTSQ-s			
Hyperglycaemia frequency perception	3.00 [2.00; 4.00]	3.00 [2.00; 4.00]	0.385
Hypoglycaemia frequency perception	2.00 [2.00; 3.00]	3.00 [2.00; 4.00]	0.022
Final score	28.00 [23.00; 31.00]	28.00 [24.00; 31.80]	0.987

Data are shown as median [interquartile]. AWI, average weighted impact score; DR, diabetic retinopathy; QoL, quality of life.

**Table 3:** Multivariable linear regression models for present quality of life, work life and dependence as reported on the Audit of Diabetes Dependent Quality of Life (ADDQoL-19) questionnaire.

	Present QoL ^1^		Work Life ^2^		Dependence ^3^	
	Estimate β (95% CI)	*p*	Estimate β (95% CI)	*p*	Estimate β (95% CI)	*p*
(Intercept)	1.00 (0.83; 1.17)	<0.001	−1.81 (−2.31; −1.32)	<0.001	−2.45 (−1.51; −1.73)	<0.001
Retinopathy	−0.25 (−0.49; −0.01)	0.040	−0.78 (−1.50; −0.05)	0.036	−0.83 (−1.51; −0.16)	0.016
Smoker, current	0.12 (−0.14; 0.39)	0.361	0.07 (−0.74; 0.87)	0.867	0.49 (−0.26; 1.24)	0.197

No significant contribution of the variables according to the likelihood ratio test: age, sex, race, educational level, insulin dose, physical activity, hypertension, dyslipidaemia, diabetes duration, body mass index, waist circumference, microalbuminuria, glycated haemoglobin, systolic and diastolic blood pressure, HDL and LDL cholesterol and triglycerides. Model estimates (β) refers to the overall mean (intercept) and the estimated change in the score mean between patient with and without the corresponding characteristic. ^1^ Coefficient of determination, r-squared: 2.0%. ^2^ Coefficient of determination, r-squared: 2.3%. ^3^ Coefficient of determination, r-squared: 3.8%. QoL, quality of life.

**Table 4:** Multivariable linear regression model for hypoglycaemia frequency perception of the Diabetes Treatment Satisfaction Questionnaire-status (DTSQ-s).

	Estimate β (95% CI)	*p*
(Intercept)	2.91 (2.62; 3.20)	<0.001
Retinopathy	0.44 (0.07; 0.80)	0.019
Sex, male	−0.60 (−0.96; −0.24)	0.001

No significant contribution of the variables according to the likelihood ratio test: age, race, educational level, insulin dose, physical activity, hypertension, dyslipidaemia, diabetes duration, body mass index, waist circumference, smoking, systolic and diastolic blood pressure, triglycerides, glycated haemoglobin, microalbuminuria, HDL and LDL cholesterol. Model estimates (β) refers to the overall mean (intercept) and the estimated change in the score mean between patient with and without the corresponding characteristic. Coefficient of determination, *r*-squared: 6.3%.

## Supplementary Materials

The following are available online at https://www.mdpi.com/2077-0383/8/3/377/s1, Table S1: Multivariate linear regression for the Audit of Diabetes Dependent Quality of Life (ADDQoL-19) average weighted impact score, Table S2: Multivariate linear regression for the Diabetes Treatment Satisfaction Questionnaire-status (DTSQ-s) final score.
