# Supplementary material for: Poorer Quality of Life and Treatment Satisfaction is Associated with Diabetic Retinopathy in Patients with Type 1 Diabetes without Other Advanced Late Complications
Source: J Clin Med. 2019 Mar 18;8(3):377. doi: 10.3390/jcm8030377 (PMC6462963; doi:10.3390/jcm8030377)
Supplement: Supplementary file 1 [file jcm-08-00377-s001.pdf]

Table S1. Multivariable linear regression for the Audit of Diabetes Dependent Quality of Life (ADDQoL-19) average weighted impact score.

	**Estimate (95% CI)**	***p*-Value**
(Intercept)	–1.50 (–2.32; –0.69)	<0.001
Retinopathy	–0.29 (–0.66; 0.07)	0.111
Age (years)	–0.02 (–0.04; 0.00)	0.015
Physical activity	0.58 (0.18; 0.99)	0.005
Smoker, current	–0.14 (–0.53; 0.25)	0.481
Insulin dose	0.01 (0.00; 0.02)	0.028

No significant contribution of the variables according to the likelihood ratio test: sex, race, educational level, hypertension, dyslipidaemia, diabetes duration, body mass index, waist circumference, systolic and diastolic blood pressure, triglycerides, glycated haemoglobin, microalbuminuria, HDL and LDL cholesterol. Coefficient of determination, r-squared = 7.4%.

Table S2. Bivariate analysis for the Audit of Diabetes Dependent Quality of Life (ADDQoL–19) and the Diabetes Treatment Satisfaction Questionnaire–status (DTSQ–s) by diabetic retinopathy status.

Items	No DR (n = 140)	DR: Grade 1 (n = 83)	DR: Grade 2–4 (n = 19)	p-Value		
				No DR vs DR: Grade 1	No DR vs. DR: Grade 2–4	p Overall
**ADDQoL–19**						
Present QoL	1.00 [1.00; 2.00]	1.00 [0.00; 1.00]	1.00 [0.00; 1.00]	0.040	0.304	0.068
Diabetes–specific QoL	–1.00 [–2.00; –1.00]	–2.00 [–2.00; –1.00]	–2.00 [–2.00; –1.00]	0.119	0.188	0.272
Leisure	–1.00 [–3.00; 0.00]	–2.00 [–3.00; 0.00]	–2.00 [–4.00; 0.00]	0.194	0.536	0.377
Work life	0.00 [–3.00; 0.00]	–2.00 [–4.00; 0.00]	–3.00 [–6.00; 0.00]	0.100	0.083	0.087
Travels	–2.00 [–4.00; 0.00]	–1.00 [–4.00; 0.00]	0.00 [–2.00; 0.00]	0.667	0.086	0.520
Holidays	–2.00 [–3.00; 0.00]	–2.00 [–3.00; 0.00]	0.00 [–4.00; 0.00]	0.794	0.652	0.914
Physical ability	–2.00 [–4.00; 0.00]	–2.00 [–4.00; 0.00]	–2.00 [–3.00; 0.00]	0.143	0.858	0.542
Family life	0.00 [–3.00; 0.00]	0.00 [–3.00; 0.00]	0.00 [–3.00; 0.00]	0.407	0.481	0.645
Friends/social life	0.00 [0.00; 0.00]	0.00 [0.00; 0.00]	0.00 [0.00; 0.00]	0.546	0.782	0.968
Personal relationship	0.00 [–2.00; 0.00]	0.00 [–2.00; 0.00]	0.00 [–3.00; 0.00]	0.187	0.245	0.450
Sex life	0.00 [–2.00; 0.00]	0.00 [–3.00; 0.00]	0.00 [–3.00; 0.00]	0.121	0.111	0.074
Physical appearance	0.00 [–1.00; 0.00]	0.00 [–2.00; 0.00]	0.00 [–1.00; 0.00]	0.056	0.995	0.166
Self–confidence	0.00 [–2.25; 0.00]	0.00 [–4.00; 0.00]	0.00 [–3.00; 0.00]	0.234	0.837	0.398
Motivation	0.00 [–2.00; 0.00]	0.00 [–2.00; 0.00]	0.00 [–2.00; 0.00]	0.970	0.852	0.598
Society/people's reaction	0.00 [0.00; 0.00]	0.00 [0.00; 0.00]	0.00 [0.00; 0.00]	0.535	0.247	0.472
Future	–2.00 [–4.00; 0.00]	–2.00 [–6.00; 0.00]	–2.00 [–6.00; 0.00]	0.206	0.300	0.389
Finances	0.00 [0.00; 0.00]	0.00 [0.00; 0.00]	0.00 [0.00; 0.00]	0.135	0.137	0.223
Living conditions	0.00 [0.00; 0.00]	0.00 [0.00; 0.00]	0.00 [0.00; 0.00]	0.475	0.422	0.423
Dependence	0.00 [–3.00; 0.00]	–2.00 [–4.00; 0.00]	–2.00 [–4.00; 0.00]	0.005	0.320	0.055
Freedom to eat	–4.00 [–6.00; –2.00]	–6.00 [–9.00; –2.00]	–4.00 [–9.00; –2.00]	0.407	0.786	0.625
Freedom to drink	–2.00 [–4.50; 0.00]	–4.00 [–7.00; 0.00]	–2.00 [–6.00; 0.00]	0.117	0.858	0.231
AWI	–1.32 [–2.05; –0.68]	–1.60 [–2.70; –1.00]	–1.50 [–2.50; –0.90]	0.038	0.372	0.186
**DTSQ–s**						
Hyperglycaemia frequency perception	3.00 [2.00; 4.00]	3.00 [2.00; 4.00]	3.00 [3.00; 4.00]	0.683	0.253	0.386
Hypoglycaemia frequency perception	2.00 [2.00; 3.00]	3.00 [2.00; 4.00]	3.00 [2.00; 4.00]	0.098	0.032	0.051
Final score	28.00 [23.00; 31.00]	27.00 [24.00; 31.00]	29.00 [25.00; 32.00]	0.530	0.316	0.486

Data are shown as median [interquartile]. AWI, average weighted impact score; DR, diabetic retinopathy; QoL, quality of life. Grade 1: mild diabetic retinopathy; Grade 2–4: moderate, severe and proliferative diabetic retinopathy.

Table S3. Multivariable linear regression for the Diabetes Treatment Satisfaction Questionnaire-status (DTSQ-s) final score.

	**Estimate (95% CI)**	***p*-Value**
(Intercept)	27.72 (25.45; 29.98)	<0.001
Retinopathy	–0.91 (–2.46; 0.63)	0.244
Smoker, current	0.37 (–1.20; 1.93)	0.643
Diabetes duration if women	–0.02 (–0.11; 0.07)	0.716
Diabetes duration if men	0.24 (0.13; 0.36)	<0.001
Men if diabetes duration > 20 years	0.39 (–1.03; 1.80)	0.588

No significant contribution of the variables according to the likelihood ratio test: age, sex, race, educational level, insulin dose, physical activity, hypertension, dyslipidaemia, body mass index, waist circumference, systolic and diastolic blood pressure, triglycerides, glycated haemoglobin, microalbuminuria, HDL and LDL cholesterol. Coefficient of determination, *r*–squared: 7.5%. The interaction between sex and diabetes duration was statistically significant with a *p*-value of 0.0003.
